# MHD Mixed Convective Peristaltic Motion of Nanofluid with Joule Heating and Thermophoresis Effects

**DOI:** 10.1371/journal.pone.0111417

**Published:** 2014-11-12

**Authors:** Sabir Ali Shehzad, Fahad Munir Abbasi, Tasawar Hayat, Fuad Alsaadi

**Affiliations:** 1 Department of Mathematics, Comsats Institute of Information Technology, Sahiwal, Pakistan; 2 Department of Mathematics, Quaid-I-Azam University, Islamabad, Pakistan; 3 Communication Systems and Networks (CSN) Research Group, Department of Electrical and Computer Engineering, Faculty of Engineering, King Abdulaziz University, Jeddah, Saudi Arabia; China University of Mining and Technology, China

## Abstract

The primary objective of present investigation is to introduce the novel aspect of thermophoresis in the mixed convective peristaltic transport of viscous nanofluid. Viscous dissipation and Joule heating are also taken into account. Problem is modeled using the lubrication approach. Resulting system of equations is solved numerically. Effects of sundry parameters on the velocity, temperature, concentration of nanoparticles and heat and mass transfer rates at the wall are studied through graphs. It is noted that the concentration of nanoparticles near the boundaries is enhanced for larger thermophoresis parameter. However reverse situation is observed for an increase in the value of Brownian motion parameter. Further, the mass transfer rate at the wall significantly decreases when Brownian motion parameter is assigned higher values.

## Introduction

Mixed convection in vertical channels is of considerable importance for the enhancement of cooling systems in engineering. This includes modern heat exchangers, nuclear reactors, solar cells and many other electronic devices. Such flows are mainly affected by buoyancy. MHD mixed convective heat transfer analysis in vertical channels is of considerable importance due to its applications in self-cooled or separately cooled liquid metal blankets, cooling systems for electronic devices, solar energy collection and chemical processes. Use of nanoparticles as means to enhance the heat transfer in low thermal conductivity fluids has proven to be a novel technique [1]. Addition of nanoparticles has proven to facilitate the heat transfer phenomena by enhancing the thermal conductivity of low thermal conductivity fluids e.g. water, engine oil etc. Presently the nanofluids are largely used in medical, engineering and electrical processes. Such fluids find applications in hyperthermia, cryosurgery, modern drug delivery systems, heating and cooling systems, batteries of electronic devices, etc. Wide utility of nanofluids has paved the way for a new science named nanofluid mechanics. The science of nanofluids mechanics has gained considerable attention of motivated researchers from all over the world (see refs. [2]–[5]). Recently, Turkyilmazoglu [6] investigated the heat and mass transfer of MHD slip flow in nanofluids. In this problem the author computed the exact solutions of the involved equations. Natural convective boundary layer flow of nanofluid past a vertical surface is studied by Rashidi et al. [7]. Turkyilmazoglu [8] obtained the analytical solution for MHD mixed convection heat transfer flow of viscoelastic fluid over a stretching surface. Sheikholeslami et al. [9] investigated the nanofluid filled enclosure in presence of magnetic field with heat flux boundary. Turkyilmazoglu [10] analyzed the nanofluid flow and heat transfer due to rotating disk. Rashidi et al. [11] reported the second law analysis in flow of nanofluid induced by a rotating disk. Sheikholeslami and Ganji [12] studied the magnetohydrodynamic flow of viscous nanofluid in a permeable channel.

Peristaltic pumping is a mechanism of the fluid transport in a flexible tube by a progressive wave of contraction or expansion from a region of lower pressure to higher pressure. Peristalsis is one of the major mechanisms for fluid transport in physiology. It is an involuntary and key mechanism that moves food through the digestive tract, bile from the gallbladder into the duodenum, transport of blood through the artery with mild stenosis, urine from the kidneys through the ureters into the bladder and sperm through male reproductive track. Further, several engineering appliances including roller and finger pumps, hose pumps, dialysis and heart-lung machines are designed on the principle of peristalsis. Subject to such extensive applications, several investigators studied peristaltic flows under different flow configurations [13]–[17]. Recent developments in hyperthermia, cryosurgery and laser therapy as means to destroy the undesirable tissues in cancer therapy has triggered great interest in the analysis of peristaltic flows with heat transfer (see refs. [18]–[21]). Peristaltic transport of nanofluids is significant in modern drug delivery systems and cancer therapy. Review of the available literature indicates that studies focusing the peristaltic transport of nanofluids are scarce. Some of the available studies for peristaltic transport of nanofluids can be seen through refs. [22]–[24].

It is noticed that none of the above mentioned studies and others in the existing literature investigate the novel aspect of peristaltic transport with the thermophoresis boundary condition. This study aims to fill this void. Therefore this article investigates the peristaltic transport of viscous nanofluid under the influence of constant applied magnetic field. Incompressible fluid is taken in a channel with thermophoresis condition at the boundaries [4], [5]. Mixed convection, viscous dissipation and Joule heating are also taken into account. Mathematical modelling is carried out using the long wavelength and low Reynolds number approximations. Resulting coupled equations are numerically solved using Mathematica 8.0. Graphical analysis is carried out to examine the influence of various embedded parameters on the velocity, temperature, concentration of nanoparticles and heat and mass transfer rates at the wall.

## Mathematical Analysis

Consider a viscous nanofluid in two-dimensional symmetric channel of width 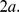 Walls of the channel are maintained at constant temperature 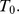 A uniform magnetic field of strength 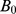 is applied in a direction normal to the channel walls. Effects of induced magnetic field are discarded under the assumption of low magnetic Reynolds number. Cartesian coordinate system is defined in such a manner that the 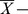
axis lies along the length of channel and the 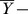
axis is normal to the 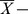
axis. Sinusoidal waves with amplitude 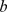 and wavelength 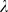 propagate along the channel walls in the 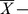
direction with constant speed 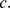 Flow within the channel is induced due to the propagation of such waves. Geometry of the peristaltic walls is given below:
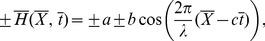
(1)where 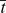 is time and 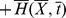 and 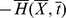 are the walls lying in the positive and negative 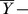
direction respectively. Velocity field for the two-dimensional flow in Cartesian coordinate system is given by 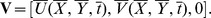 Law of conservation of mass, linear momentum, energy and concentration are given below:
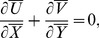
(2)

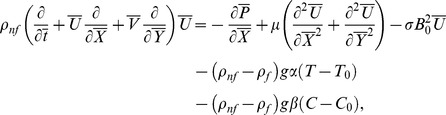
(3)

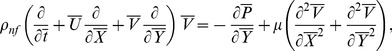
(4)

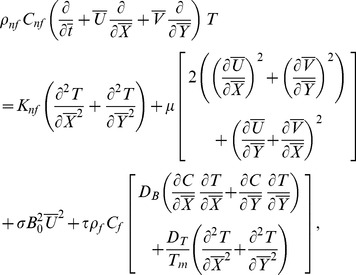
(5)

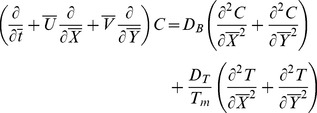
(6)


Here 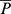 is the pressure, 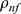 the density of nanofluid, 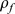 the density of base fluid, 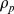 the density of nanoparticles, 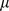 the dynamic viscosity of nanofluid, 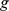 the acceleration due to gravity, 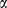 the thermal expansion coefficient, 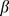 the concentration expansion coefficient, 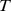 the fluid temperature, 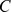 the concentration, 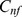 the specific heat of nanofluid, 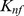 the thermal conductivity of nanofluid, 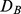 the mass diffusivity, 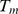 the mean temperature and 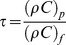 where 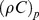 designates the effective heat capacity of nanoparticles. We transform our problem into a frame of reference (wave frame) moving along the wave with same speed. The quantities from the fixed frame 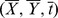 to wave frame 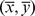 are related by the following transformations:
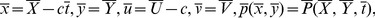
(7)in which 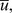 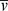 and 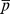 are the velocity components and pressure in wave frame 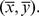 Transformed set of equations takes the form
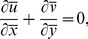
(8)

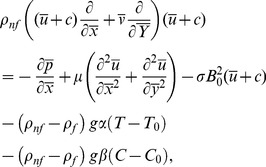
(9)

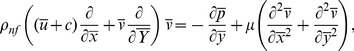
(10)

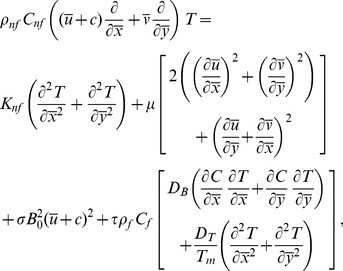
(11)

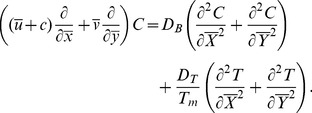
(12)


We define the following dimensionless quantities in order to non-dimensionalize the problem
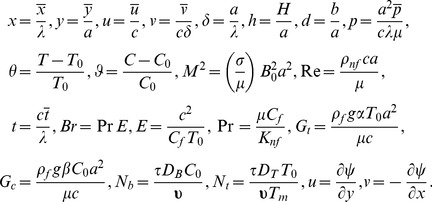
(13)


The assumptions of long wavelength and small Reynolds number give 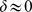 and 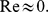 It should be pointed out that the theory of long wavelength and zero Reynolds number remains applicable for case of chyme transport in small intestine [25]. In this case *c = 2* cm/min, 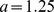 cm and 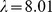 cm. Here half width of intestine is small in comparison to wavelength. i.e. 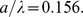 It is also declared by Lew et al. [26] that Reynolds number in small intestine was small. Further, the situation of intrauterine fluid flow due to myometrial contractions is a peristaltic type fluid motion in a cavity. The sagittal cross section of the uterus reveals a narrow channel enclosed by two fairly parallel walls [27]. The 1–3 mm width of this channel is very small compared with its 50 mm length [28], defining an opening angle from cervix to fundus of about 0.04 rad. Analysis of dynamics parameters of the uterus revealed frequency, wavelength, amplitude and velocity of the fluid-wall interface during a typical contractile wave were found to be 0.01–0.057 Hz, 10–30 mm, 0.05–0.2 mm and 0.5–1.9 mm/s. Applying the long wavelength and low Reynolds number approximations [25]–[30] the Eqs. (9–12) in view of Eq. (13) are reduced to
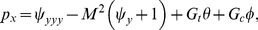
(14)

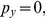
(15)

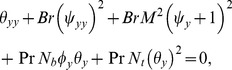
(16)

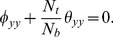
(17)


Here 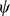 denotes the stream function, 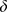 the wave number, 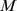 the Hartman number, 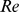 the Reynolds number, 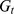 the thermal Grashoff number, 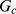 the concentration Grashoff number, 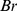 the Brinkman number, 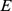 the Eckret number, 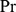 the Prandtl number, 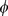 dimensionless concentration, 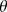 the dimensionless temperature, 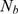 the Brownian motion parameter, 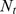 the thermophoresis parameter and as a result of long wavelength and low Reynolds number approximations pressure becomes independent of transverse coordinate which can be seen through Eq. (15). the continuity equation is identically satisfied. Cross differentiation of Eqs. (14) and (15) gives
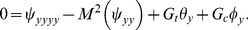
(18)


Defining 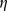 and *F* as the dimensionless mean flows in the laboratory and wave frames by
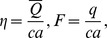
one can write
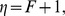
where
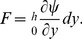
(19)


Kuznetsov and Nield [4], [5] proposed that instead of considering constant concentration of nanoparticles at the boundaries, it is more realistic to take into account the thermophoresis at the boundaries. Hence we also assume that in presence of thermophoresis the normal fluxes of nanoparticles is zero at the boundaries. Mathematically, this condition is given as
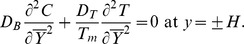



Thus the dimensionless boundary conditions in the present flow are
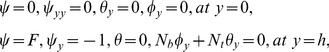
(20)where 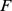 is defined in Eq. (19) and 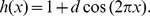


The system of Eqs. (16–18) subject to boundary conditions Eq. (20) is solved numerically through NDSolve in Mathematica. Obtained numerical results are analyzed graphically in the next section.

## Discussion

This portion of the article aims to analyze the numerical results through graphs. Plots for axial velocity, temperature and concentration are prepared and analyzed. Heat and mass transfer rates at the wall are studied through bar-charts.

### Analysis of axial velocity

**Figure 1:**
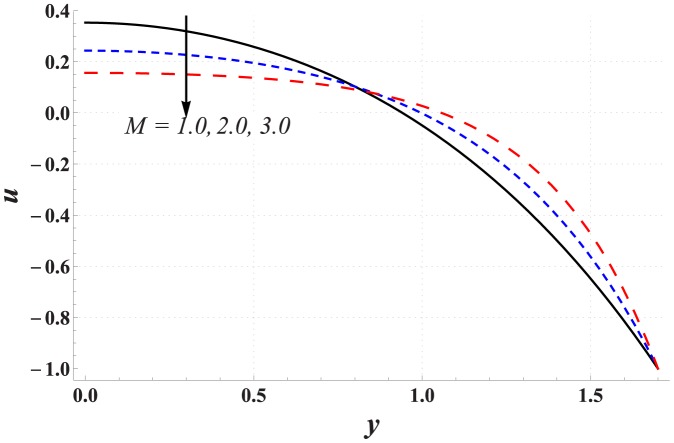
Effect of Hartman number on the velocity of nanofluid when 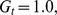 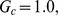 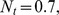 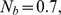 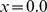, 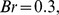 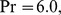 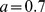 and 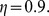

**Figure 2:**
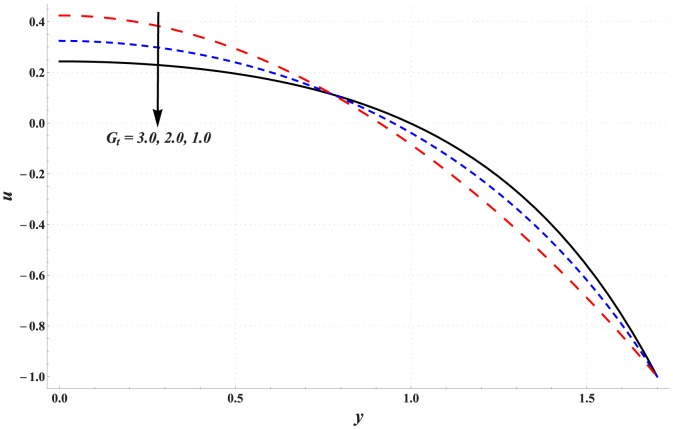
Effect of thermal Grashoff number on the velocity of nanofluid when 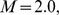 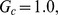 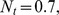 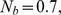 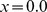, 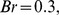 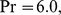 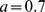 and 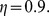

**Figure 3:**
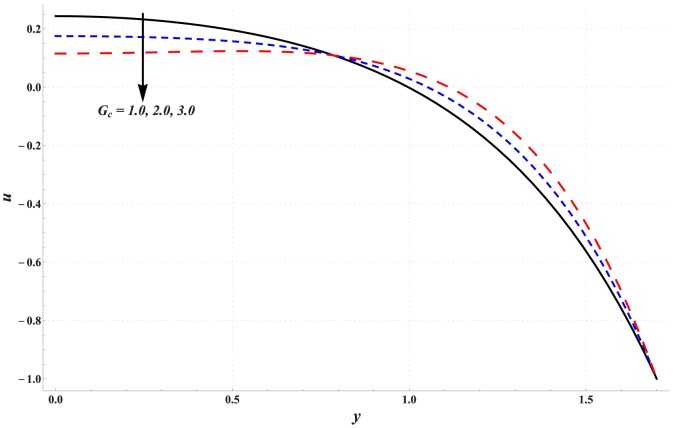
Effect of concentration Grashoff number on the velocity of nanofluid when 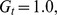 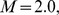 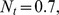 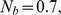 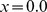, 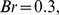 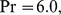 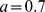 and 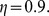

**Figure 4:**
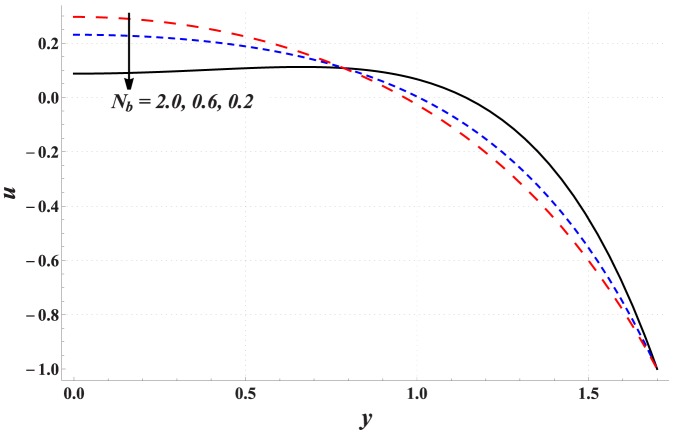
Effect of Brownian motion parameter on the velocity of nanofluid when 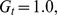 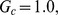 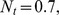 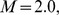 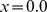, 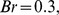 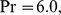 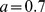 and 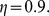

**Figure 5:**
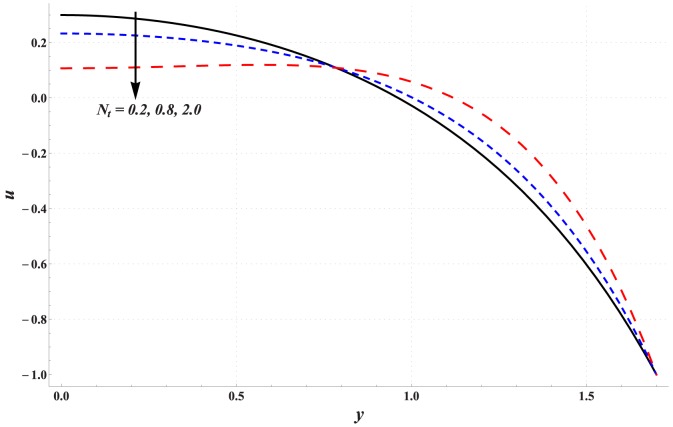
Effect of thermophoresis parameter on the velocity of nanofluid when 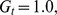 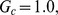 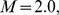 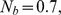 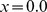, 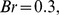 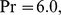 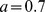 and 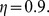

Figs. 1–5 are prepared to examine the influences of various parameters on velocity profile. All these Figs. show that velocity attains maximum value near the centre of channel. Also it can be seen from these Figs. that effects of different parameters on velocity are opposite near the channel walls compared with that of near the centre of channel. Fig. 1 depicts that velocity near the centre of channel decreases by increasing Hartman number. This signifies the reduction of maximum velocity subject to an increase in the strength of applied magnetic field. Thermal Grashoff number has an increasing effect of the velocity near the centre (see Fig. 2). Substantial increase in the maximum velocity is seen for an increase in thermal Grashoff number. Contrary to previous case, maximum velocity decreases when concentration Grashoff number is increased (see Fig. 3). Brownian motion and thermophoresis parameters have opposite effects on the velocity (see Figs. 4 and 5). Maximum velocity is found to increase when Brownian motion parameter is enhanced.

### Analysis of temperature

**Figure 6:**
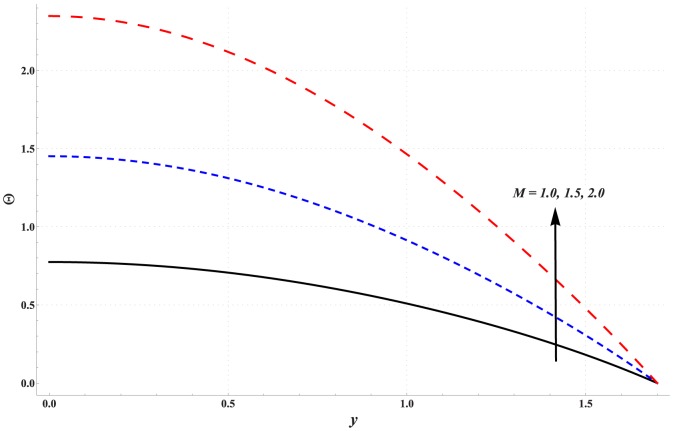
Effect of Hartman number on the temperature profile when 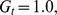 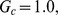 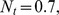 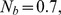 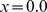, 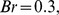 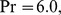 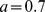 and 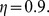

**Figure 7:**
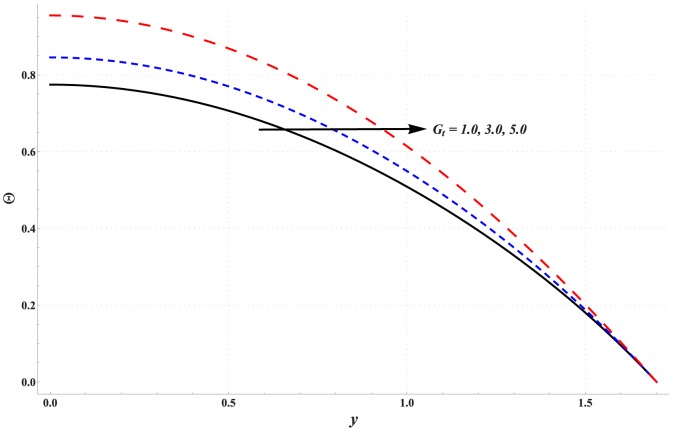
Effect of thermal Grashoff number on the temperature profile when 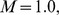 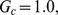 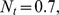 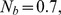 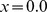, 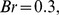 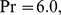 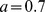 and 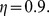

**Figure 8:**
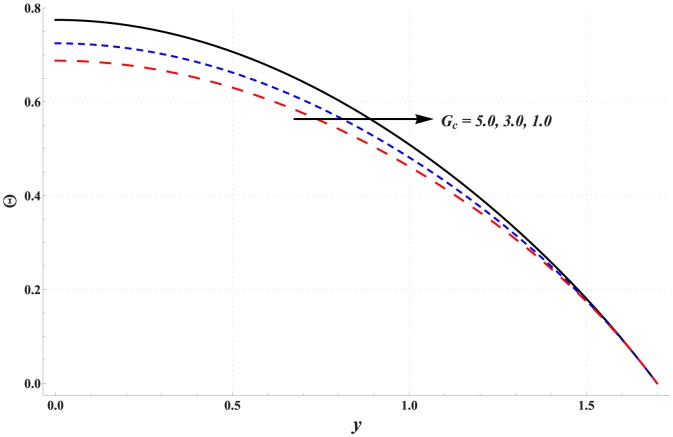
Effect of concentration Grashoff number on the temperature profile when 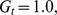 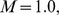 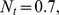 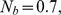 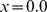, 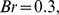 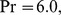 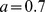 and 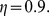

**Figure 9:**
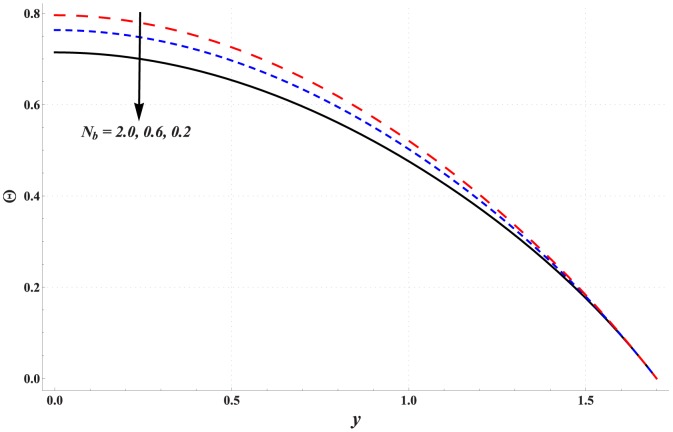
Effect of Brownian motion parameter on the temperature profile when 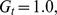 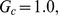 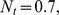 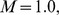 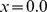, 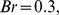 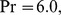 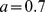 and 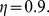

**Figure 10:**
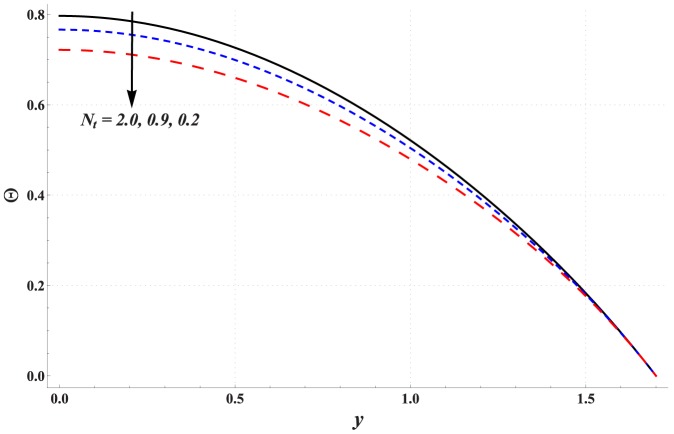
Effect of thermophoresis parameter on the temperature profile when 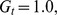 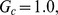 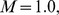 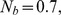 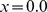, 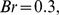 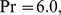 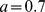 and 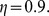

Temperature profile for variation in different parameters is studied through Figs. 6–10. Significant rise in the temperature of fluid is observed when Hartman number is increased (see Fig. 6). This fact is physically justified due to Joule heating. Fig. 7 shows that an increase in thermal Grashoff number results in an increase in temperature of fluid. Such increase in temperature however is small when compared with that of previous case. Fig. 8 indicates that increase in concentration Grashoff number slightly decreases the fluid temperature. Increase in Brownian motion parameter enhances the temperature (see Fig. 9). Such increase becomes insignificant for *Nb>1*. Fig. 10 shows a decrease in temperature when thermophoresis parameter is increased.

### Concentration of nanoparticles

**Figure 11:**
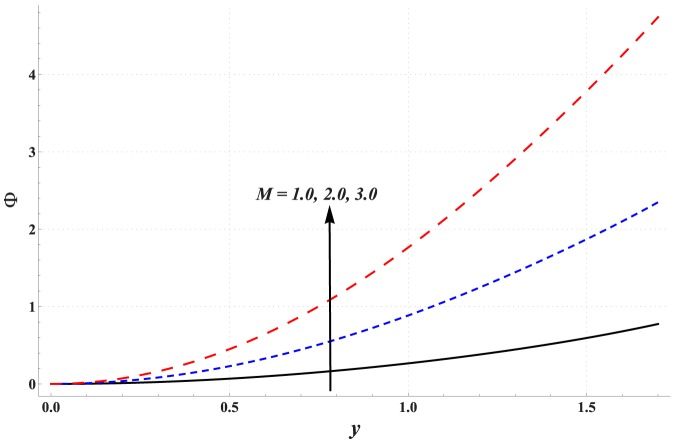
Concentration profile for variation in Hartman number when 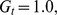 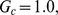 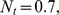 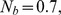 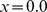, 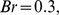 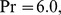 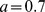 and 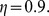

**Figure 12:**
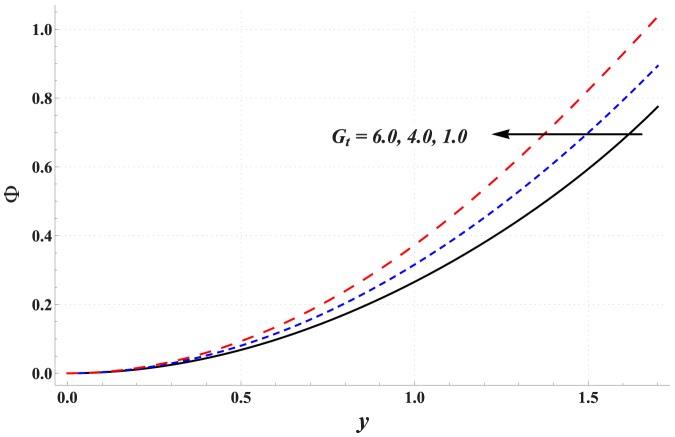
Concentration profile for variation in thermal Grashoff number when 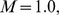 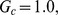 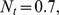 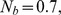 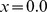, 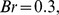 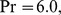 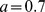 and 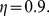

**Figure 13:**
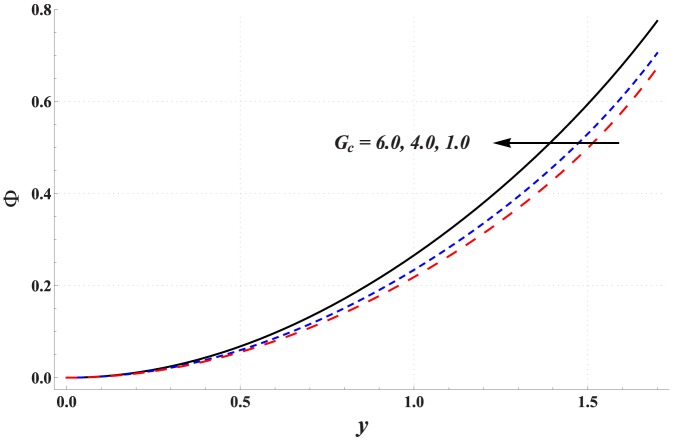
Concentration profile for variation in concentration Grashoff number when 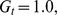 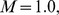 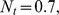 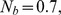 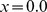, 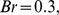 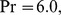 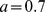 and 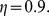

**Figure 14:**
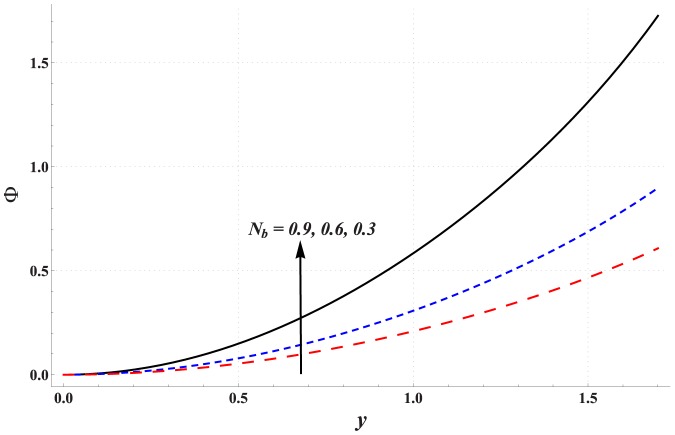
Concentration profile for variation in Brownian motion parameter when 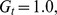 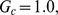 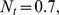 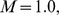 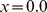, 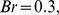 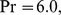 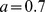 and 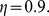

**Figure 15:**
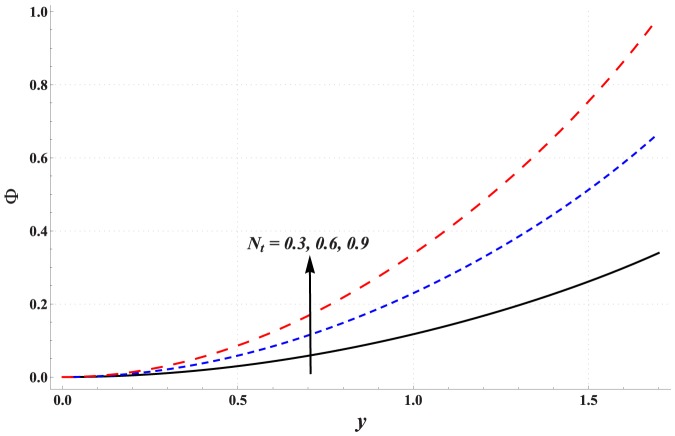
Concentration profile for variation in thermophoresis parameter when 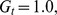 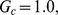 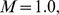 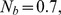 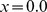, 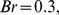 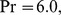 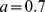 and 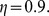

**Figure 16:**
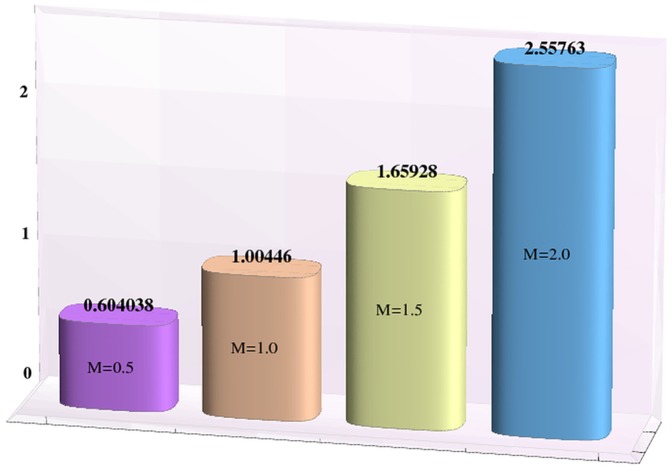
Effect of Hartman number on the heat transfer rate the wall 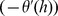 when 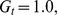 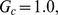 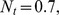 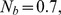 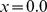, 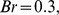 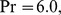 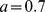 and 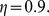

Impact of different parameters on the concentration of nanoparticles is examined through Fig. 11–16. These graphs indicate that the concentration of nanoparticles near the channel walls is largely influenced by the change in embedded parameters. This is mainly due to consideration of the thermophoresis condition for concentration. A common observation from these Figs. is that the impact of a parameter on concentration of nanoparticles is proportional to the impact of that parameter on fluid temperature. Fig. 11 depicts that the concentration of nanoparticles considerably increases with an increase in Hartman number. This is mainly the consequence of Joule heating and the thermophoresis boundary condition. Concentration of the nanoparticles enhances with an increase in the value of thermal Grashoff number (see Fig. 12). Contrary to this case the concentration of nanoparticles decreases with an increase in the value of concentration Grashoff number (see Fig. 13). The Brownian motion and thermophoresis parameters have opposite effects on the concentration of nanoparticles (see Figs. 14 & 15). Considerable increase in the concentration of nanoparticles is reported when the value of thermophoresis parameter increases. Such increase becomes large as we move from the center of channel towards the channel walls.

### Heat and mass transfer rates

**Figure 17:**
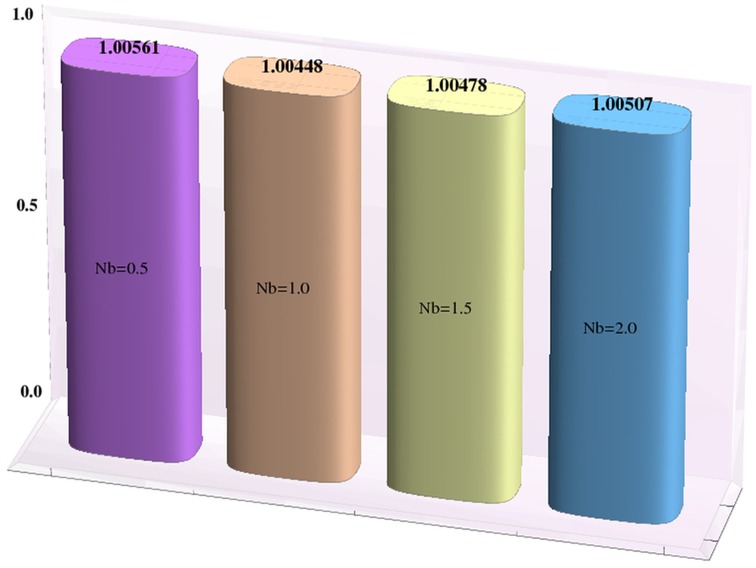
Effect of Brownian motion parameter on the heat transfer rate the wall 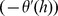 when 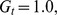 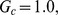 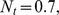 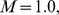 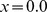, 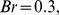 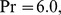 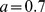 and 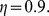

**Figure 18:**
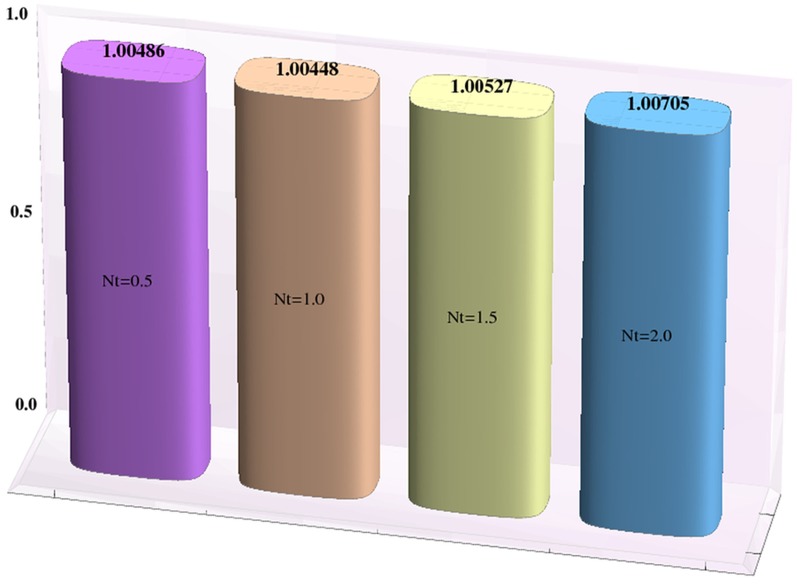
Effect of thermophoresis parameter on the heat transfer rate the wall 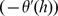 when 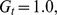 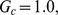 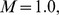 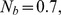 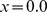, 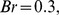 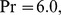 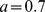 and 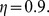

**Figure 19:**
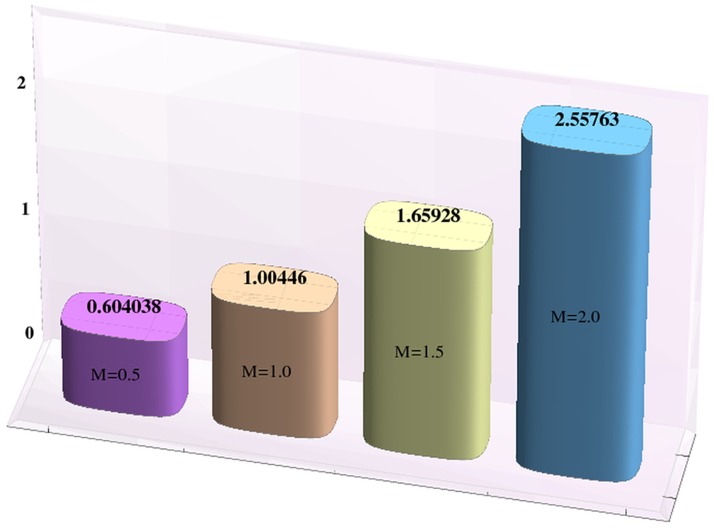
Effect of Hartman number on the mass transfer rate the wall 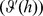 when 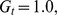 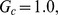 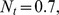 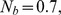 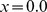, 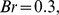 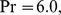 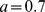 and 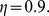

**Figure 20:**
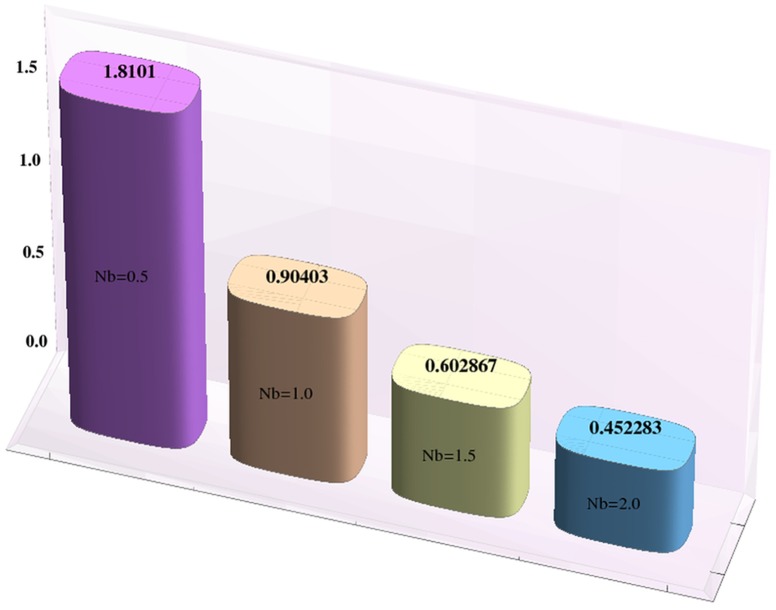
Effect of Brownian motion parameter on the mass transfer rate the wall 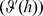 when 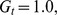 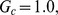 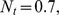 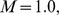 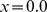, 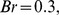 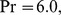 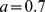 and 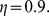

**Figure 21:**
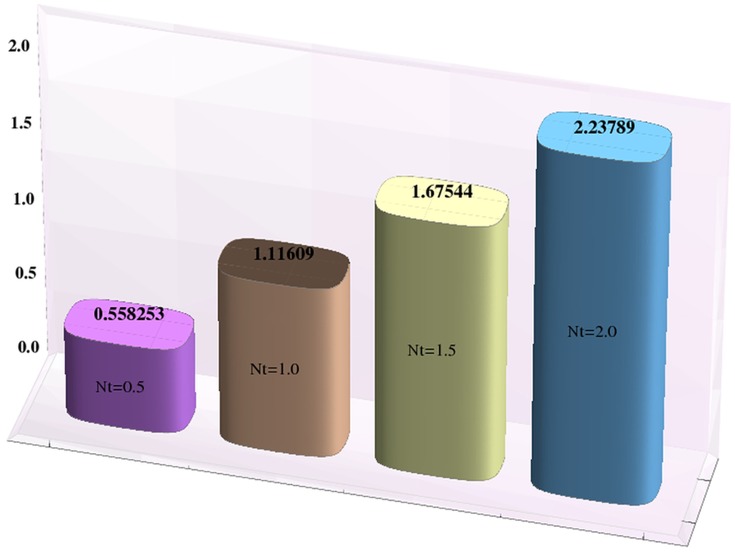
Effect of thermophoresis parameter on the mass transfer rate the wall 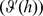 when 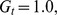 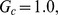 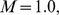 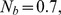 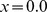, 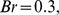 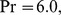 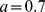 and 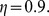

Effects of Hartman number, Brownian motion and thermophoresis parameters on the heat and mass transfer rates at the wall are studied through bar-charts given in Figs. 16–21. Considerable increase in the heat transfer rate at the wall is noted when the strength of applied magnetic field is increased (see Fig. 16). The Brownian motion and thermophoresis parameters have very little effect on heat transfer rate at the wall (see Figs. 17–18). This is primarily due to the fact that we have assumed the channels walls to possess constant temperature. Heat transfer rate at the wall slightly increases with an increase in Brownian motion parameter. However this observation is not true for 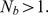 Fig. 19 depicts that the mass transfer rate at the wall significantly increases with an increase in the value of Hartman number. This is primarily due to the effect of applied magnetic field. The Brownian motion and thermophoresis parameters have opposite effects on the temperature of fluid. Considerable decrease in mass transfer rate at the wall is seen for an increase in Brownian motion parameter. This is the consequence of considered thermophoresis condition for concentration.

### Comparison with available results

In order to check the validity of the solution methodology and the obtained results, we provide the comparison of the special case of present study with the results of Ali et al. [13]. Comparison between the numerical values of pressure rise per wavelength obtained through exact solutions by Ali et al. [13] is provided with that of the special case of present study (see Table 1). It is seen that the two results are in good agreement which confirms the validity of present results.

**Table 1:** Comparison of the critical values of flow rate below which 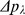 is positive and above which it is negative computed by Ali et al. [13] and limiting case of present study.

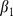	Ali et al. [13]	Present study
0.0	0.3683	0.368529
0.03	0.3533	0.353565
0.06	0.3411	0.341398

## Conclusions

MHD mixed convective peristaltic transport of viscous nanofluid in a channel with thermophoresis at the boundaries is examined. Key findings of this study are summarized below.

- Thermal and concentration Grashoff numbers have opposite effects on the axial velocity and temperature of nanofluid.
- Axial velocity decreases with an increase in the value of Brownian motion parameter whereas opposite is reported for thermophoresis parameter.
- Temperature of the nanofluid decreases with increase in the strength of applied magnetic field and Brownian motion of nanoparticles.
- Increase in the strength of applied magnetic field results in an increase in nanoparticles concentration near the channel walls.
- The Brownian motion and thermophoresis parameters have large but opposite effects on the concentration of nanoparticles.
- Significant decrease in the mass transfer rate at the wall is noted when Brownian motion parameter is assigned higher values.
